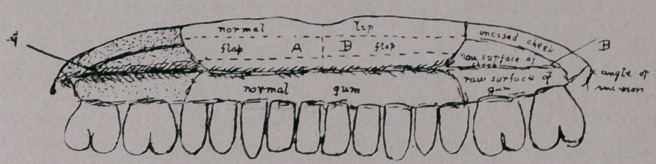# Three Cases of Closure of Jaws, Produced by Mercurial Stomatitis*Read at Waco Meeting Texas State Medical Association, April 26, 1900.

**Published:** 1900-05

**Authors:** J. E. Thompson

**Affiliations:** Galveston, Texas


For Texas Medical Journal.

### Three Cases of Closure of Jaws, Produced by Mercurial Stomatitis.*

BY J. E. THOMPSON, M. D., GALVESTON, TEXAS.

The destructive effects of mercury on the buccal cavity are so often seen in the malarial districts of our State that I need no apology for reporting three cases of cicatricial closure of the jaws that have come under my care for surgical treatment. All three cases were serious. In two, such extensive destruction of the mucous membrane of the gums and cheeks had occurred that the resulting cicatrisation had obliterated the cheek pouch and welded the upper and lower jaws together, preventing any opening of the mouth, and necessitating feeding entirely by liquids or soft finely divided solids. The third case had resulted in gangrene of the cheek (cancreun oris) and cicatricial welding of the upper and lower jaws.

CASE I.—G. L., aged 8, was brought to me from the interior on October 28th, 1895. A clear history of mercurial salivation when she was four years old was obtained.

Her condition was very pitiful. She was absolutely unable to depress the lower jaw even to the slightest degree. Her diet was mainly of liquids, but she had for some time succeeded in pushing some solid food into her mouth by main force. This act had depressed the lower incisors, so that they pointed almost horizontally backwards, while the upper incisors pointed forwards with a considerable slant.

On passing the finger between the lip and gum of both jaws one found the space normal as far out as the level of the second bicuspid teeth of right and left sides. Beyond this the space was obliterated, the cheek and gum being united by dense cicatricial tissue. The lateral boundaries of this space closely corresponds with the angles of the mouth.

*Operation.*—October 31st, on each side, an incision was made through the cheek from the angle of the mouth directly backwards to the anterior edge of the masseter. The soft parts were cut through and the cicatricial tissue divided. A gag was inserted and the mouth widely opened. No difficulty was experienced, for it did not appear that there was any ankylosis in the temporo-maxillary articulation.

---

*Read at Waco Meeting Texas State Medical Association, April 26, 1900.

As far back as the level of the last molar teeth the cicatricial tissue and cheek were dissected from the outer side of the alveolar process. This was done on both sides, above and below.

From the normal part of the upper lip in front a long rectangular flap of mucous membrane was cut. The free end of this was at the median line and the attached end at the level of the angle of the mouth. It was swung around on its outer attachment and carefully united by sutures to the inner surface of the cheek (which had been dissected from the alveolus) in such a way that its mucous lining faced the raw alveolus, and the raw surface faced the cheek flap. This procedure was repeated on the other side, and then similar flaps were cut from the lower lip. The free edges of both right flaps were united at their apices, and likewise those of both left flaps. The edges of the raw surfaces on the lips were united, after which the primary incisions through the cheeks were carefully sutured. Horsehair sutures were used throughout.

Healing was most satisfactory. In seven days an anæsthetic was given and a gag was inserted and the jaws opened slightly. After this, at intervals of a few days, the gag was used. Very soon we had the co-operation of the child, and she was accustomed from time to time during the day to depress the lower jaw with her fingers. She soon began to chew solids, and was discharged on December 17th, cured. At this time she could separate her front teeth fully three quarters of an inch, and eat like any other child.

Since this date I have heard that there has been no relapse.

Case II.—C. H. P., aged 7; native of Louisiana. Came under my care on March 19th, 1899, suffering from the effects of mercurial stomatitis. Mercurialism occurred when he was two years old.

The symptoms were almost identical with those of Case I, but there had been some destruction of the mucous membrane of both upper and lower lips in front, diminishing the depth of the space between them and the gum.

The same operation was done as in Case I, but owing to the lack of mucous membrane the flaps were much narrower than they ought to have been. They were placed as described in Case I, and healing occurred by first intention. The result was good, but owing to the narrowness of the flap, it was not so good as I expected. The patient was discharged on April 5th, with an interval of half an inch between upper and lower incisor teeth and perfect ability to eat solid food.

Case III.—M. L., aged 11; native Texan. Came under my care on December 12th, 1899, suffering from the effects of mercurial stomatitis, the result of treatment a few months previously.

The jaws were completely closed, and the boy had been fed ever since the salivation on fluid food. The breath was very fœtid, and the gums still quite spongy. On the left cheek there was a hole the size of half a dollar, through which the molar teeth of both upper and lower jaws could be seen.

The patient was kept under observation for some weeks, until the fœtor abated somewhat, and on January 8th, a triangular piece of bone was removed from the body of the jaw just in front of the masseter muscle. The base of the triangle was at the lower border, and measured about half an inch. The apex was about one-fourth inch wide, and corresponded with the socket of the second molar tooth. The incision was carried from the hole in the cheek to the lower margin of the jaw, and the bone sawed through with a chain saw. It was necessary to remove almost all the molar and bicuspid teeth of both sides, because they were hanging almost unattached in diseased sockets.

The result was very satisfactory. The jaw could be depressed fully an inch and a quarter, all fœtor disappeared, and the hole in the face closed up rapidly. It became as small as a dime, but contraction beyond this was impossible, so I operated in the following way, which I have found very successful in closing fistulous tracts:

A circular incision was made about one-fourth inch from the margin, and the skin dissected up almost to the edge. It was then inverted through the opening, and the raw surface of the circular flap brought into contact by a purse string suture of catgut. From the side of the face a flap of skin was cut and made to slide on its attached pedicle over the raw surface. Horsehair sutures closed the wounds thus made.

The result was perfect. On the fourth day the horsehair stitches were removed and healing was complete.

The patient was discharged in a very satisfactory condition.

## Figures and Tables

**Figure f1:**